# Supplementary material for: Comprehensive Physiologically Based Pharmacokinetic Model to Assess Drug–Drug Interactions of Phenytoin
Source: Pharmaceutics. 2023 Oct 18;15(10):2486. doi: 10.3390/pharmaceutics15102486 (PMC10609929; doi:10.3390/pharmaceutics15102486)
Supplement: Supplementary file 1 [file pharmaceutics-15-02486-s001.zip › pharmaceutics-2592644-supplementary.pdf]

Comprehensive Physiologically Based Pharmacokinetic Model to Assess Drug–Drug Interactions of Phenytoin

Supplementary Materials

Leyanis Rodriguez-Vera [1,†], Xuefen Yin [1,†], Mohammed Almoslem [1], Karolin Romahn [1], Brian Cicali [1], Viera Lukacova [2], Rodrigo Cristofoletti [1,*] and Stephan Schmidt [1,*]

[1] Center for Pharmacometrics and System Pharmacology at Lake Nona (Orlando), Department of Pharmaceutics, College of Pharmacy, University of Florida, Orlando, FL 32827, USA; leyanisrv80@gmail.com (L.R.-V.); xuefenyin@ufl.edu (X.Y.); almoslem.mj@gmail.com (M.A.); karolinromahn@gmail.com (K.R.); bcicali@ufl.edu (B.C.)
[2] Simulations Plus, Lancaster, CA 93534, USA; viera.lukacova@simulations-plus.com
[*] Correspondence: rcristofoletti@ufl.edu (R.C.) and sschmidt@cop.ufl.edu (S.S.)
[†] These authors contributed equally to this work.

Contents

List of Tables

S1 Initial PBPK Model - Simulated *versus* observed PK parameters for phenytoin 250, 300, 400, and 900 mg intravenous and oral single and multiple doses in healthy subjects--3

S2 Solubility vs. pH profile from Serajuddin et al. 1993, and Chiang et al. 2013----------------------------------5

S3 Dose proportionality evaluation results in the range 200-900 mg after single and multiple i.v. and oral doses--6

S4 Drug-dependent parameters of the drugs used in DDIs PBPK model for phenytoin------------------------6

S5 DDI dynamic simulation- Simulated *versus* observed PK parameters ratio for phenytoin as a victim with fluconazole (200 and 400 mg), omeprazole (NM and IM), and phenytoin as a perpetrator with itraconazole in healthy subjects---7

List of Figures

S1 Local sensitivity analysis --8

S2 Mass balance of phenytoin by Caraco et al. 2001---8

S3 Relationship of phenytoin AUC values versus dose using the dose proportionality power model ------9

S4 Model prediction of phenytoin concentration-time profiles of different studies in comparison with the observed data. After 250, 300, 400, and 900 mg single dose, in different formulations and administration routes (i.v. infusion and oral), in comparison with the observed data---10

S5 Model prediction of phenytoin concentration-time profiles in fast and fed conditions, after 300 mg single dose, tablet formulation, in comparison to observed data--11

S6 Simulated and observed phenytoin DDIs in semilogarithmic scale--12

S7 Phenytoin exposures and changes in the pharmacokinetic parameters in different unbound fraction (fup) scenarios---13

Table S1. Physiologically-based pharmacokinetic (PBPK) Model - Simulated *versus* observed PK parameters for phenytoin 250, 300, 400, and 900 mg intravenous and oral single and multiple doses in healthy subjects.

Dose [mg]	Route	N Gender	Age [years]	Weight [kg]	Data set	C_{max} (Sim) [µg/mL]	C_{max} (Obs) [µg/mL]	C_{max} ratio (Sim/Obs)	AUC $_{0\text{-}t}$ (Sim)(µg-h/mL)	AUC $_{0\text{-}t}$ (Obs) [µg-h/mL]	$AUC_{0\text{-}t}$ ratio (Sim/Obs)	AUC $_{0\text{-}\infty}$ (Sim) [µg-h/mL]	AUC $_{0\text{-}\infty}$ (Obs) [µg-h/mL]	$AUC_{0\text{-}\infty}$ ratio (Sim/Obs)	Reference
250 mg SD	i.v. infusion (5min)	6 M	26-48	75 [70.8-78.2]	Training[a]	-	-	-	115.94	121.77	**0.95**	118.54	137.0	**0.86**	Glazko et al. 1969 [1]
300 mg SD	po	18M	30.5 [22-43]	-	Training[b]	4.19	3.22	**1.30**	137.32	118.55	**1.16**	140.64	123.77	**1.14**	Caraco et al. 2001 [2]
300 mg SD	po	6M	-	78.2 [71.4-88.2]	Training[c]	3.95	3.83	**1.03**	126.05	116.57	**1.08**	135.47	131.37	**1.03**	Gugler et al. 1976 [3]
300 mg MD	po	6M	-	78.2 [71.4-88.2]	Training[d]	7.94	8.15	**0.93**	252.18	267.32	**0.94**	270.47	290.79	**0.93**	Gugler et al. 1976 [3]
300 mg SD	i.v. infusion (6min)	6 M	-	78.2 [71.4-88.2]	Test	-	-	-	131.38	164.1	**0.80**	140.12	175.63	**0.80**	Gugler et al. 1976 [3]
300 mg SD	po	32M	27.6 [21-40]	77.4	Test	4.66	4.35	**1.07**	134.4	141.89	**0.95**	137.96	144.29	**0.96**	Ducharme et al. 1995 [4]
300 mg SD	po	10M	19-25	63-92	Test	4.06		**1.12**	125.92		**0.97**	138.34		**1.05**	Prichard et al. 1987 [5]

						3.64		129.29		131.5		
400 mg SD	po	6M	24-54	59-74	Test	4.74 / 3.72	**1.27**	148.89 / 136.09	**1.09**	180.11 / 188.99	**0.95**	Dill et al. 1956 [6]
900 mg SD	po	6M	27.2 [22-32]	74.7 [68.2-82.7]	Test	5.44 / 8.83	**0.61**	213.6 / 322.73	**0.66**	300.6 / 446.08	**0.67**	Fraser et al. 1980 [7]
300 mg SD,	po Fasted	1 F 7 M	23-27	61-80	Test	4.11 / 4.56	**0.90**	115.11 / 115.89	**0.99**	139.03 / 133.17	**1.04**	Melander et al. 1979 [8]
300 mg[a] SD,	po Fed	1 F 7 M	23-27	61-80	Test	4.82 / 6.32	**0.76**	117.62 / 132.39	**0.89**	140.82 / 146.41	**0.96**	Melander et 1979 [8]
250 mg SD	po, tablet	11M	30.2 [22-42]	-	Test	3.70 / 3.18	**1.16**	112.8 / 108.1	**1.04**	115.6 / 109	**1.06**	Smith et al. 1976 [9]
248 mg SD	po, capsule	11M	30.2 [22-42]	-	Test	3.64 / 2.72	**1.34**	111.8 / 99.18	**1.13**	114.6 / 101.6	**1.13**	Smith et al. 1976 [9]
200 mg _MD+ 250 mg SD	po for 3 days + i.v. infusion (10 min)	20M	19-43	-	Test	- / -	**-**	113.05 / 123.95	**0.91**	192.36 / 202.72	**0.95**	Blum et al. 1991 [10]
250 mg SD	po	9M	22-35	-	Test	3.76 / 4.26	**0.88**	99.69 / 215.79	**0.46**	115.83 / 274.89	**0.42**	Touchette et al. 1992 [11]
N=163 subjects						GMFE (range)	1.01 (0.76- 1.34)		0.91 (0.46-1.16)		0.91 (0.42-1.14)	
						Sim/Obs within 2- fold	12 /12		14 /15		14/15	

a: the clinical study used for establishing distribution phase; b: the clinical study used for establishing metabolism phase; c: the clinical study used for establishing the absorption phase, and explore nonlinearity behavior; d: clinical study after multiple doses for stablishing autoinduction mechanism. (-) No available. The setting of integration time Step was "one-tenth hour". Cmax: maximum concentration; AUC: area under the curve; Sim: simulated; Obs: observed; SD: single dose; MD: multiple dose; i.v. intravenous; po: oral administration; GMFE: geometric mean fold error

Table S2. Solubility vs. pH profile from Serajuddin et al. 1993 [12], and Chiang et al. 2013 [13]

PH	Solubility (mg/mL)
1.06	0.04
3.29	0.04
4.0	0.03
4.99	0.02
6.0	0.03
6.0	0.04
6.48	0.03
7.93	0.07
7.99	0.04
8.58	0.12
8.88	0.2
8.97	0.26
8.99	0.25
9.03	0.22
9.23	0.39
9.52	0.72
9.67	1.12
9.81	0.78
9.81	1.85
10.02	2.0
10.04	2.65
10.3	4.27
10.3	5.19
10.51	6.31
10.52	9.73
10.71	9.33

Table S3. Dose proportionality evaluation results in the range 200-900 mg after single and multiple i.v. and oral doses

	Dose range	Beta	90% CI lower bound	90% CI higher bound	BE limits (0.8 - 1.25)
**i.v. SD**	200-900 mg	1.3264	1.1704	1.4823	0.8516-1.1483
**i.v. SD***	200-600 mg	1.0339	1.0058	1.062	0.7969-1.2031
**i.v. MD**	200-900 mg	1.1084	1.0733	1.1435	0.8516-1.1483
**oral SD**	200-900 mg	0.5314	0.3503	0.7526	0.8516-1.1483
**oral MD**	200-900 mg	0.6911	0.4793	0.903	0.8516-1.1483

*Re-evaluation of dose linearity removing the 900 mg dose demonstrating that dose proportionality in the range 200-600 mg.

Red color highlight values outside the BE limits indicating lack of dose proportionality

Table S4. Drug-dependent parameters of the drugs used in drug-drug interactions (DDIs) PBPK model for phenytoin

Drug	DDI Mechanisms	Parameters	Unit	Value	Reference
Fluconazole		MW	g/mol	306.3	GastroPlus library
		logP	-	0.4	GastroPlus library
		pKa	-	2.03	GastroPlus library
		R_{bp}	-	1	GastroPlus library
	Competitive Inhibition	$Ki_{rev\text{-}invivo,U}$ (CYP2C9)	µM	19.6	Kunze et al. 1996 [15]
		$Ki_{rev\text{-}invitro,U}$ (CYP2C19)	µM	1.74	Kunze et al. 1996 [15]
		fup	-	0.87	Kunze et al. 1996 [15]
Omeprazole		MW	g/mol	345.4	GastroPlus library
		logP	-	2.04	GastroPlus library
		pKa	-	14.7<br>7.1	GastroPlus library
		R_{bp}	-	0.58	GastroPlus library
		fup	-	5	GastroPlus library
	Mechanism-based Inactivation + Competitive Inhibition	$K_{I\text{-}irr\text{-}invitro,T}$ (CYP2C19)	µM	1.1	Zvyaga et al. 2012 [16]
		K_{inact} (CYP2C19)	min^{-1}	0.048	Zvyaga et al. 2012 [16]
		$IC50_{\text{-}rev\text{-}invitro,T}$ (CYP2C19)	µM	8.4	Shirisaka et al. 2013 [16]

$Ki_{rev\text{-}invivo,U}$: Reversible in vivo inhibition constant, unbound; $Ki_{rev\text{-}invitro,U}$: Reversible in vitro inhibition constant, unbound; fu: unbound fraction; $K_{I\text{-}irr\text{-}invitro,T}$: Irreversible in vitro concentration for half-maximal inactivation, total; K_{inact}: maximum inactivation rate constant; $IC50_{\text{-}rev\text{-}invitro,T}$: Reversible half maximal inhibitory concentration

Table S5. DDI dynamic simulation- Simulated *versus* observed PK parameters ratio for phenytoin as a victim with fluconazole (200 and 400 mg), omeprazole (NM and IM), and phenytoin as a perpetrator with itraconazole in healthy subjects.

Reference	Dosing	$C_{max\ ratio}$ (DDI/ Baseline) Simulated	$C_{max\ ratio}$ (DDI/ Baseline) Observed	DDI Cmax ratio (Simulated/ Observed)	AUC $_{0\text{-}t\ ratio}$ (DDI/ Baseline) Simulated	AUC $_{0\text{-}t\ ratio}$ (DDI/ Baseline) Observed	DDI AUC $_{0\text{-}t}$ ratio (Simulated/ Observed)	AUC $_{0\text{-}\infty\ ratio}$ (DDI/ Baseline) Simulated	AUC $_{0\text{-}\infty\ ratio}$ (DDI/ Baseline) Observed	DDI AUC $_{0\text{-}\infty}$ ratio (Simulated/ Observed)
**Blum et al. 1991** [10]	**Phenytoin** (victim) (250mg i.v- Day 13) + **Fluconazole** (perpetrator) (200mg daily oral - 15days) Single-dose	-	-	-	**2.05**	**1.72**	**1.19**	**5.38**	**4.30**	**1.25**
**Touchette et al. 1992** [11]	Phenytoin (victim) (250mg oral- Day 4) + Fluconazole (perpetrator) (400mg oral daily–5 Days)	**1.09**	**1.11**	**0.98**	**1.49**	**1.27**	**1.17**	**2.38**	**2.40**	**0.99**
**Prichard et al. 1987** [5]	**Phenytoin** (victim) (300 mg oral-Day 7) + **Omeprazole (NM)** (perpetrator) (40mg oral daily–9 Days)	**1.12**	**1.10**	**1.02**	**1.33**	**1.19**	**1.12**	**1.37**	**1.25**	**1.10**
	**Phenytoin** (victim) (300 mg oral-Day 7) + **Omeprazole (IM)** (perpetrator) (40mg oral daily–9 Days)	**1.14**	**1.10**	**1.04**	**1.44**	**1.19**	**1.21**	**1.54**	**1.25**	**1.23**
**Ducharme et al. 1995** [4]	**Itraconazole** (victim) 200 mg oral-Day 14) + **Phenytoin** (perpetrator) 300 mg oral daily 17 days	**0.362**	**0.141**	**2.14**	**0.0434**	**0.0536**	**0.89**	**0.049**	**0.0524**	**0.96**

DDI: drug-drug interaction; Cmax: maximum concentration; AUC: area under the curve; iv: intravenous; NM: extensive metabolizer; IM: intermediate metabolizer.

FIGURES

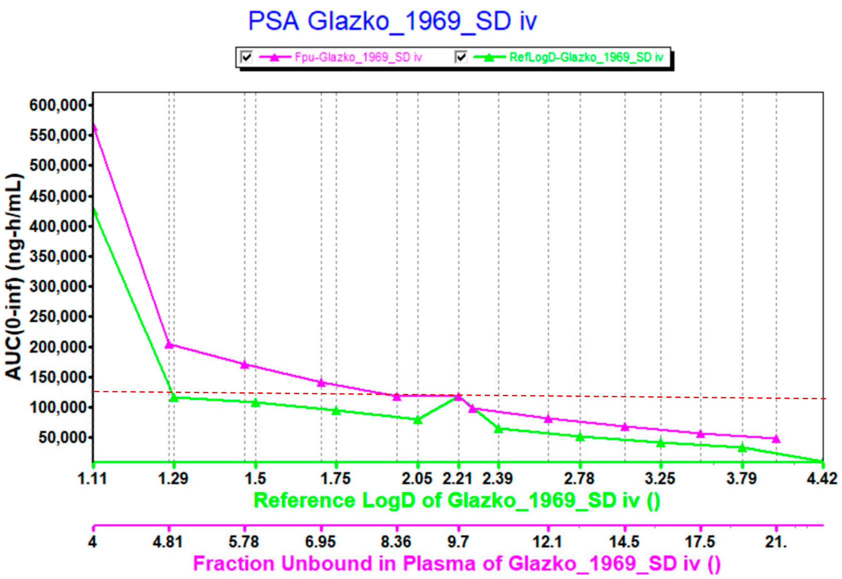

Figure S1. Local Sensitivity Analysis plot showing the unbound fraction (pink line) and lipophilicity (green line) vs. AUC0-inf from Glazko et al. 1969 clinical study. PSA: parameter sensitivity analysis

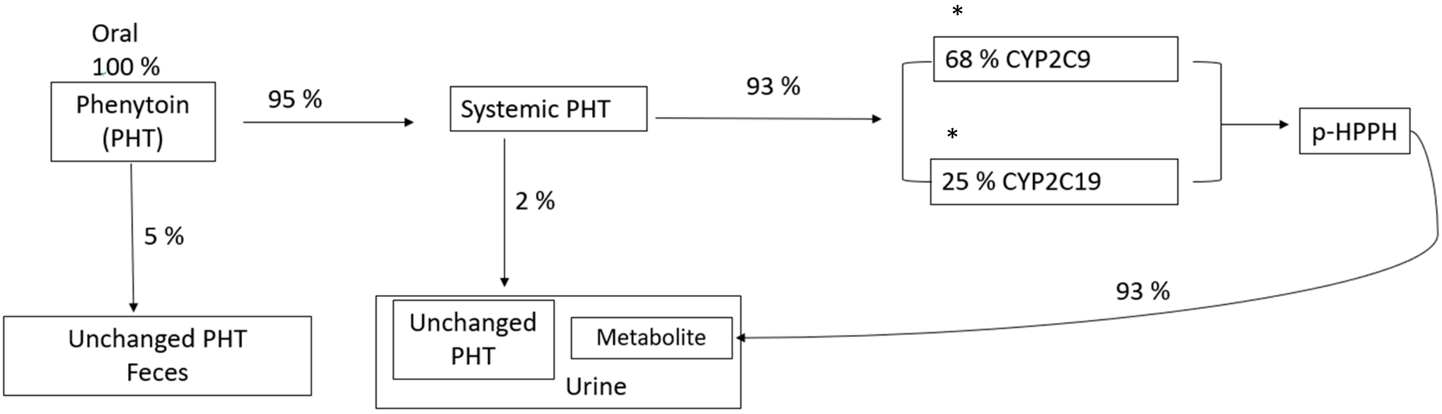

Figure S2. Mass balance of phenytoin by Caraco et al. 2001 [2]. p-HPPH: 5-parahydroxyphenyl-5-phenylhydantoin; CYP: cytochrome P450. *equivalent to 73% CYP2C9 and 27% CP2C19 of the total dose administered (100%).

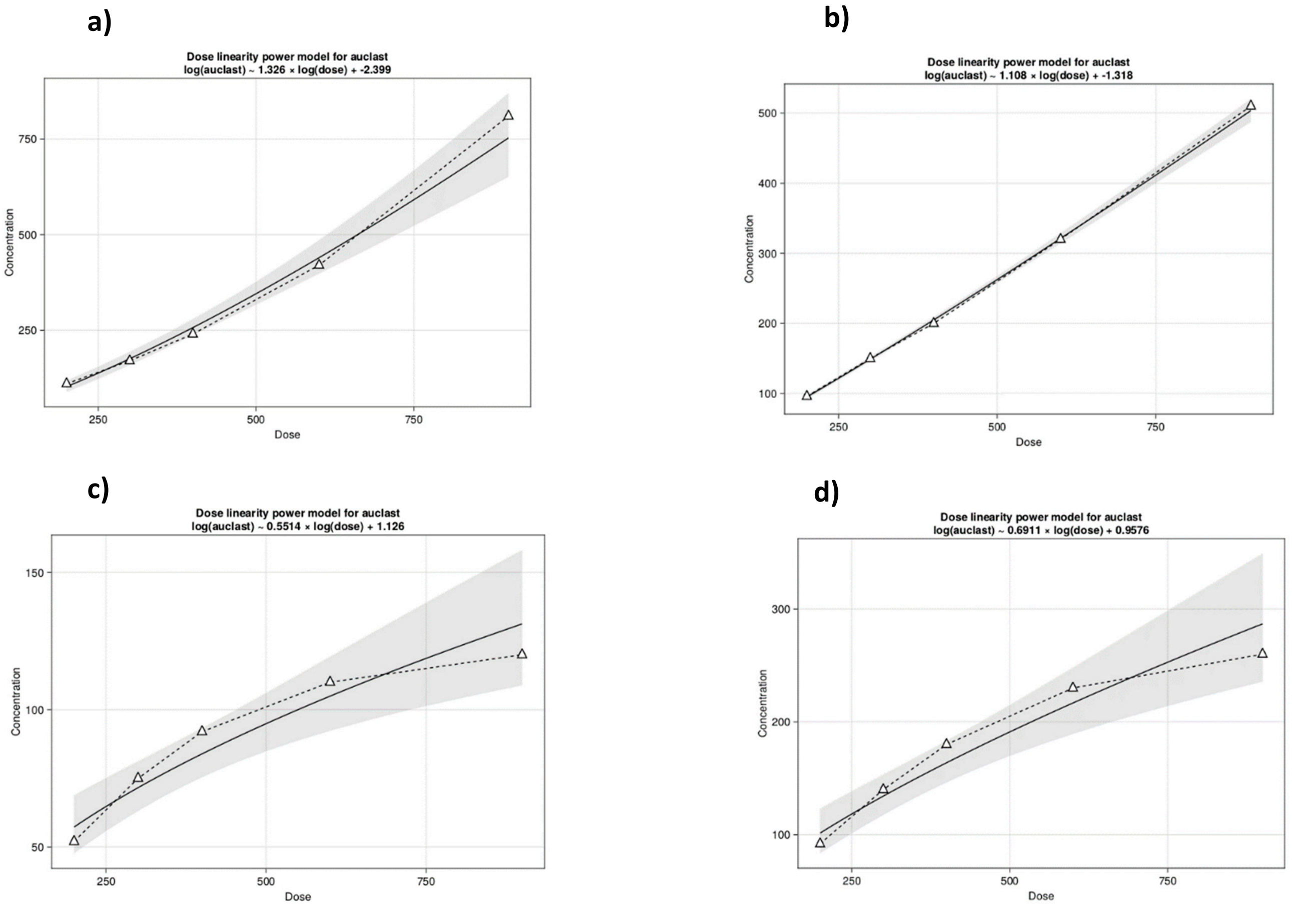

Figure S3. Relationship of phenytoin AUC values versus dose using the dose proportionality power model (*bold line*) and the 90% confidence range (*shaded area*). Triangles show the simulated values. **a)** i.v. single dose, **b)** i.v. multiple dose, **c)** oral single dose, and **d)** oral multiple dose.

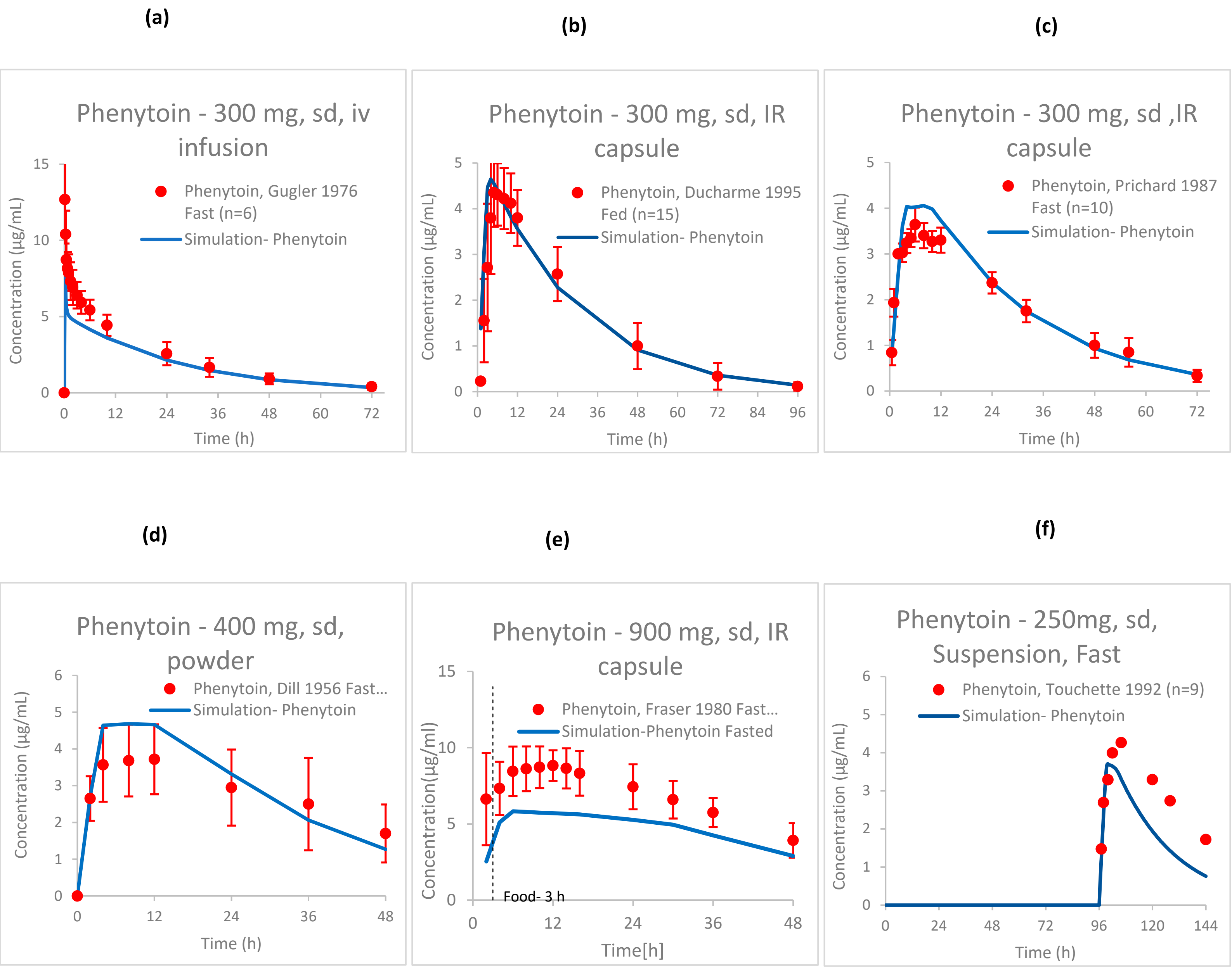

Figure S4. Model prediction of phenytoin concentration-time profiles of different studies in comparison with the observed data. After 250, 300, 400, and 900 mg single dose, in different formulations and administration routes (i.v. infusion and oral), in comparison with the observed data. Observed data are shown red dots ± Standard Deviation (if available), and simulations are shown as blue or green solid lines. sd: single dose; i.v: intravenous; IR: immediate release. * Fraser study after 3 hr was was simulated

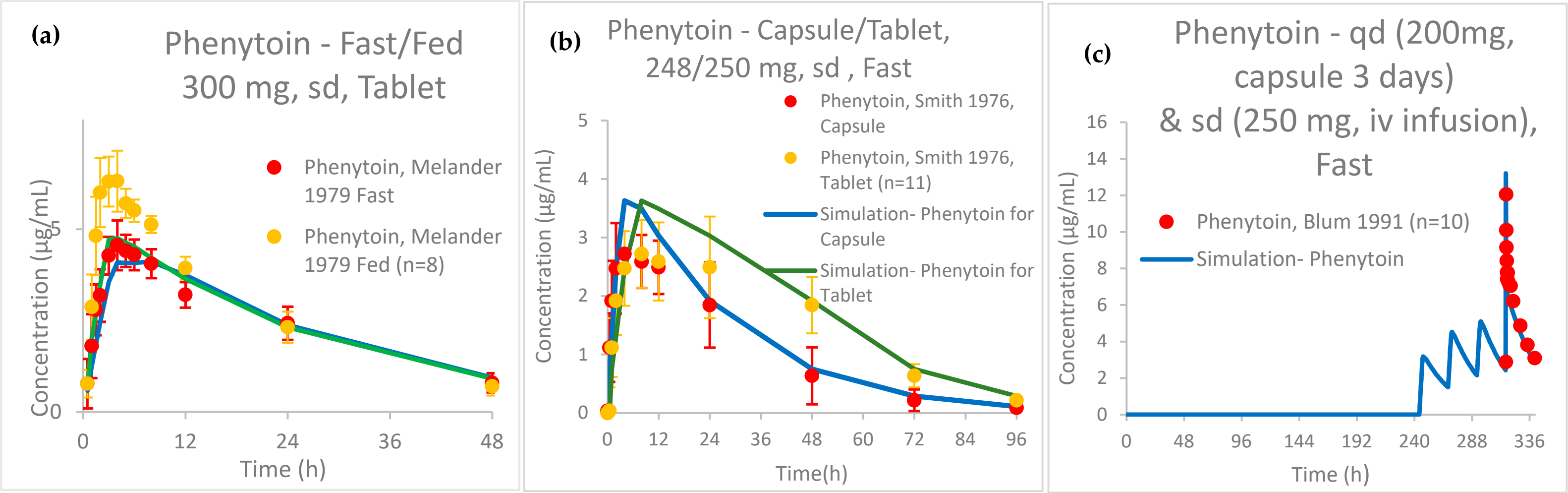

Figure S5. Model prediction of phenytoin concentration-time profiles in comparison to observed data **(a)** in fast and fed conditions, after 300 mg single dose, tablet formulation; **(b)** in different formulations: capsule and tablet and **(c)** in multiple doses of 200mg. Observed data are shown in red or yellow dots ± Standard Deviation (if available), and simulations are shown as blue or green solid lines. sd: single dose.

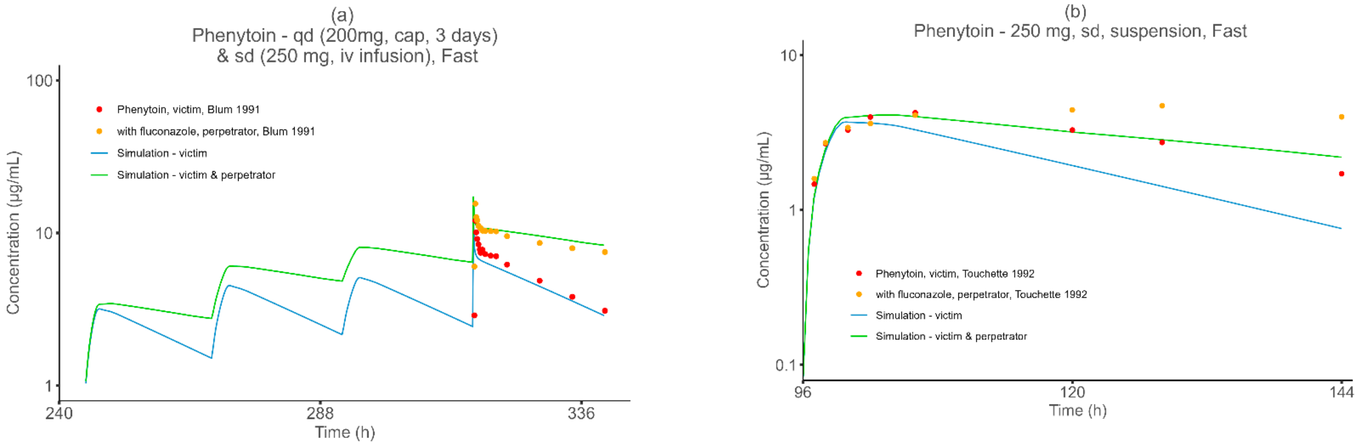

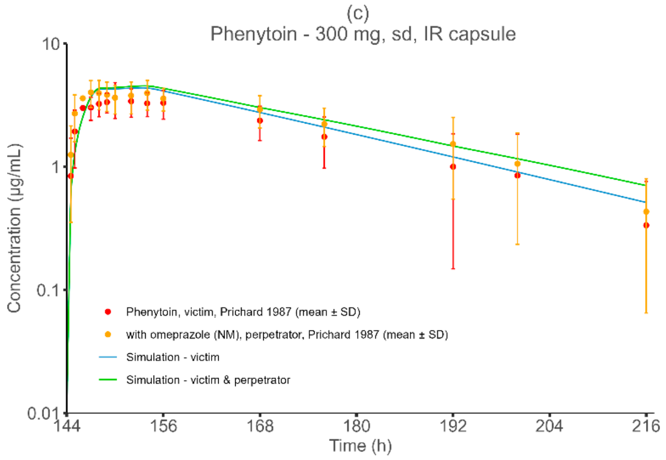

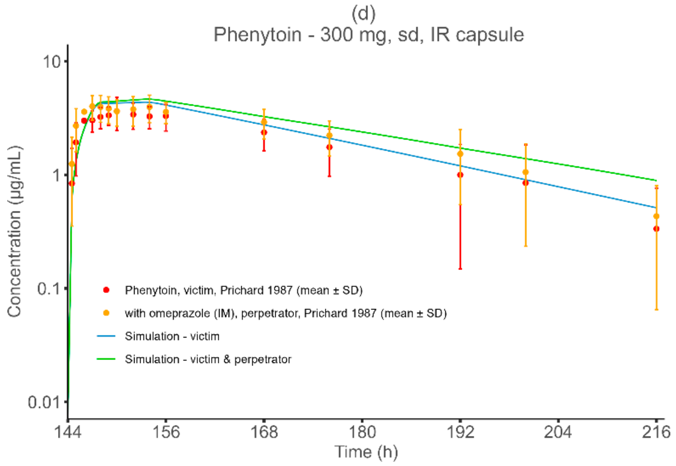

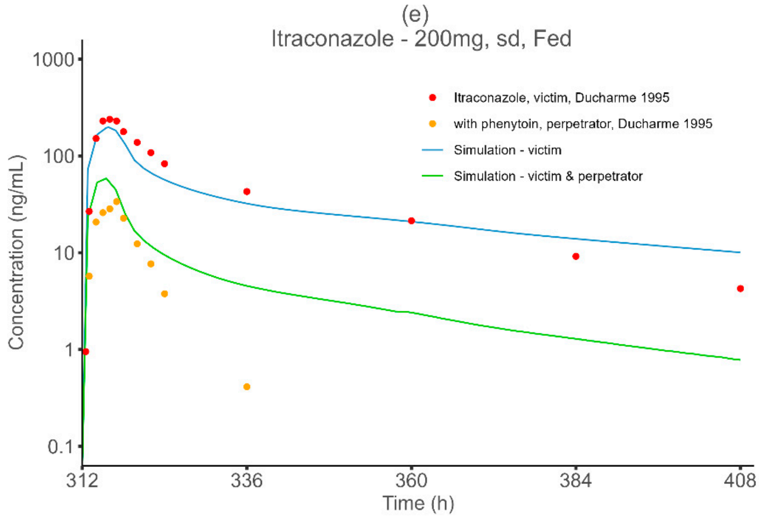

Figure S6. Simulated and observed phenytoin DDIs in semilogarithmic scale. Log Concentration-time profiles of phenytoin in comparison to observed data after **(a)** multiple- and **(b)** single-dose with and without fluconazole (200 and 400 mg) respectively, **(c)** phenytoin with and without omeprazole 40 mg in normal CYP2C19 metabolizers (NM), **(d)** phenytoin with and without omeprazole 40mg in intermediate CYP2C19 metabolizers (IM), **(e)** itraconazole concentration-times profiles before and during phenytoin co-administration. Observed data are shown as red and yellow dots, and simulations are shown as blue and green solid lines. q.d.: once daily; sd: single dose; PHT: phenytoin; IR: immediate release.

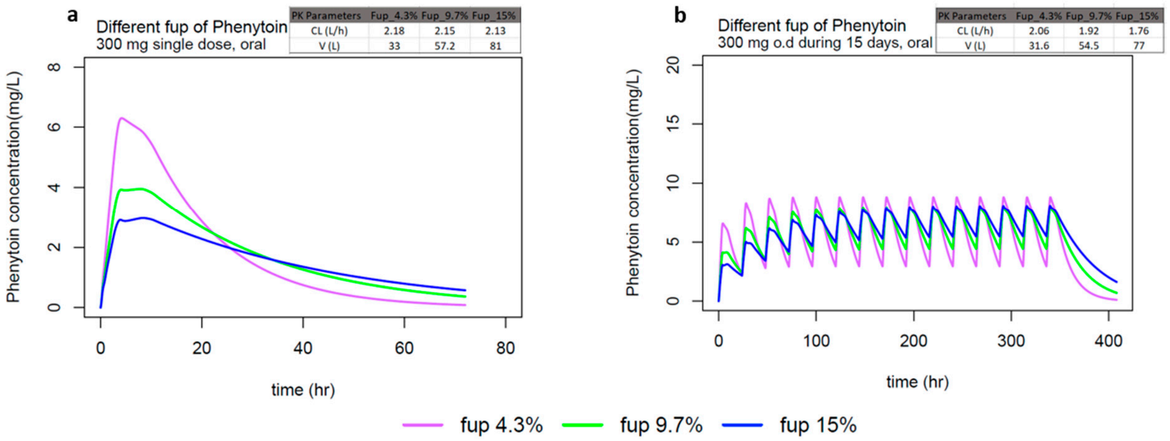

Figure S7. Phenytoin exposures and changes in the pharmacokinetic parameters in different unbound fraction (fup) scenarios after **a)** oral single dose and **b)** oral multiple dose. To account for the impact of the different fup values on the Vmax, a correction factor was calculated from the top down approach using the ratio Vmax_newfup/Vmax_fup9.7, and this correction factor was then applied to the optimized Vmax value in the final model.

References

1. Glazko, A.J.; Chang, T.; Baukema, J.; Dill, W.A.; Goulet, J.R.; Buchanan, R.A. Metabolic Disposition of Diphenylhydantoin in Normal Human Subjects Following Intravenous Administration. *Clin. Pharmacol. Ther.* **1969**, *10*, 498–504, doi:10.1002/cpt1969104498.

2. Caraco, Y.; Muszkat, M.; Wood, A.J.J. Phenytoin Metabolic Ratio: A Putative Marker of CYP2C9 Activity in Vivo. *Pharmacogenetics* **2001**, *11*, 587–596, doi:10.1097/00008571-200110000-00005.

3. Gugler, R.; Manion, C. V.; Azarnoff, D.L. Phenytoin: Pharmacokinetics and Bioavailability. *Clin. Pharmacol. Ther.* **1976**, *19*, 135–142, doi:10.1002/cpt1976192135.

4. Murray, P.; Ducharme, R.; Slaughter, L.; Warbasse, P.; Chandrasekar, V.; Edwards, D. Itraconazole and Hydroxyitraconazole Serum Concentrations Are Reduced More than Tenfold by Phenytoin. *Clin. Pharmacol. Ther.* **1995**, *58*, 617–624.

5. Prichard PJ, Walt RP, Kitchingman GK, Somervilee KW, Langman MJS, W.J. and R.A. Oral Phenytoin Pharmacokinetics during Omeprazole Therapy. *Br. J. clin. Pharmac.* **1987**, *24*, 543–545.

6. DILL, W.A.; GLAZKO, A.J.; KAZENKO, A.; WOLF, L.M. Studies on 5, 5′-Diphenylhydantoin (Dilantin) in Animals and Man. *J. Pharmacol. Exp. Ther.* **1956**, *118*, 270–279.

7. Fraser, D.G.; Ludden, T.M.; Evens, R.P.; Sutherland, E.W. Displacement of Phenytoin from Plasma Binding Sites by Salicylate. *Clin. Pharmacol. Ther.* **1980**, *27*, 165–169, doi:10.1038/clpt.1980.25.

8. Melander, A.; Brante, G.; Johansson, O.; Lindberg, T.; Wåhlin-Boll, E. Influence of Food on the Absorption of Phenytoin in Man. *Eur. J. Clin. Pharmacol.* **1979**, *15*, 269–274, doi:10.1007/BF00618516.

9. Smith, T.C.; Kinkel, A. Absorption and Metabolism of Phenytoin from Tablets and Capsules. *Clin. Pharmacol. Ther.* **1976**, *20*, 738–742, doi:10.1002/cpt1976206738.

10. Blum, R.A.; Wilton, J.H.; Hilligoss, D.M.; Gardner, M.J.; Henry, E.B.; Harrison, N.J.; Schentag, J.J. Effect of Fluconazole on the Disposition of Phenytoin. *Clin. Pharmacol. Ther.* **1991**, *49*, 420–425, doi:10.1038/clpt.1991.49.

11. Touchette, M.; Chandrasekar, P.; Milad, M.; Edwards, D. Contrasting Effects of Fluconazole and Ketoconazole on Phenytoin and Testosterone Disposition in Man. *Br. J. Clin. Pharmacol.* **1992**, *34*, 75–78, doi:10.1111/j.1365-2125.1992.tb04111.x.

12. Serajuddin, A.T.M.; Jarowski, C.I. Influence of PH on Release of Phenytoin Sodium from Slow-release Dosage Forms. *J. Pharm. Sci.* **1993**, *82*, 306–310, doi:10.1002/jps.2600820318.

13. Chiang, P.C.; Wong, H. Incorporation of Physiologically Based Pharmacokinetic Modeling in the Evaluation of Solubility Requirements for the Salt Selection Process: A Case Study Using Phenytoin. *AAPS J.* **2013**, *15*, 1109–1118, doi:10.1208/s12248-013-9519-x.

14. Wood, J.H.; Syarto, J.E.; Letterman, H. Improved Holder for Intrinsic Dissolution Rate Studies. *J. Pharm. Sci.* **1965**, *54*, 1068, doi:10.1002/JPS.2600540730.

15. Kunze, K.L.; Trager, W.F. Warfarin-Fluconazole III - A Rational Approach to Management of a Metabolically Based Drug Interaction. *Drug Metab. Dispos.* **1996**, *24*, 429–435.

16. Zvyaga, T.; Chang, S.Y.; Chen, C.; Yang, Z.; Vuppugalla, R.; Hurley, J.; Thorndike, D.; Wagner, A.; Chimalakonda, A.; Rodrigues, A.D. Evaluation of Six Proton Pump Inhibitors as Inhibitors of Various Human Cytochromes P450: Focus on Cytochrome P450 2C19. *Drug Metab. Dispos.* **2012**, *40*, 1698–1711, doi:10.1124/dmd.112.045575.
